# Mental health competencies are stronger determinants of well-being than mental disorder symptoms in both psychiatric and non-clinical samples

**DOI:** 10.1038/s41598-024-63674-9

**Published:** 2024-06-05

**Authors:** Virág Zábó, Dávid Erát, András Vargha, Ágnes Vincze, Judit Harangozó, Máté Iváncsics, Judit Farkas, Gábor Balogh, Fanni Pongrácz, Judit Bognár, Enikő Nagy, Xenia Gonda, György Purebl

**Affiliations:** 1 Doctoral School of Psychology, Eötvös Loránd University, Budapest, Hungary; 2 Faculty of Education and Psychology, Institute of Psychology, Eötvös Loránd University, Budapest, Hungary; 3 ELTE Eötvös Loránd University, Budapest, Hungary; 4 Faculty of Medicine, Institute of Behavioural Sciences, Semmelweis University, Budapest, Hungary; 5 Department of Sociology, University of Pécs, Pecs, Hungary; 6 Person- and Family-Oriented Health Science Research Group, Faculty of Humanities and Social Sciences, Institute of Psychology, Károli Gáspár University of the Reformed Church in Hungary, Budapest, Hungary; 7 National Institute of Mental Health, Neurology, and Neurosurgery, Nyírő Gyula Hospital, Budapest, Hungary; 8 Community Psychiatry Centre, Semmelweis University – Awakenings Foundation, Budapest, Hungary; 9 Department of Psychiatry and Psychotherapy, Semmelweis University, Budapest, Hungary; 10 NAP3.0-SE Neuropsychopharmacology Research Group, Hungarian Brain Research Program, Semmelweis University, Budapest, Hungary

**Keywords:** Mental health competencies, Mental disorders, Mental health test, Maintainable mental health theory, Psychiatry, Mental health promotion, Psychology, Human behaviour

## Abstract

The present study aimed to investigate whether the strength of mental health competencies and the severity of mental disorder symptoms, and their interaction, differ in the strength of their associations with several dimensions of well-being in Hungarian adult psychiatric and non-clinical samples. All respondent in the psychiatric sample (129 patients (44 male, 85 female)) and in the non-clinical community sample (253 adults (43 male, 210 female)) completed the Mental Health Test, six measures of well-being and mental health, and the Symptom Checklist-90-Revised. Including both mental health competencies and mental disorder symptoms in a regression model in both samples can predict patients’ well-being even more accurately. Mental health competencies were positively related; mental disorder symptoms were negatively related to subjective well-being. In all models and in both samples, mental health competencies were found to be stronger determinants of well-being than mental disorder symptoms. The interaction of mental health competencies and mental disorder symptoms is no more predictive of well-being in either psychiatric or non-clinical samples than when the effects of each are considered separately. The assessment of mental health competencies has an important predictive value for well-being in the presence of psychopathological symptoms and/or mental disorders.

## Introduction

In 2019, one in eight people, or 970 million individuals globally, were living with a mental disorder^1^. Consequently, understanding the impact of mental disorders on subjective well-being is crucial for psychiatric practice, research, societal attitudes, and mental health policy. Traditionally, mental disorders have been conceptualized in terms of psychopathology, focusing on the limitations arising from these conditions^2,3^. The conventional clinical recovery approach emphasizes the reduction or elimination of symptoms through standardized assessments and rigorous diagnostic criteria.

However, it is essential to shift our focus towards enhancing the subjective well-being of patients by emphasizing the possibilities of living well with mental health issues^4^. Client-centered recovery is multifaceted and predominantly subjective, prioritizing personal recovery over clinical recovery (Slade & Longden, 2015). This perspective defines recovery as achieving a personally acceptable quality of life and a positive self-perception, rather than merely the absence of symptoms. This paradigm shift allows for the consideration of strengths alongside dysfunction^5–7^ and supports individuals in leading fulfilling lives despite mental health challenges^4^. For example, Keyes’ dual continua model of mental health posits that mental health and mental illness are related but distinct dimensions, suggesting that individuals can experience high levels of well-being despite having a mental illness^8^. Additionally, studies based on Ryff’s model of psychological well-being^9^ have found that purpose in life and personal growth are positively related to well-being, even in the presence of mental disorder symptoms. The identification and application of mental health competencies in psychiatric practice, particularly during the recovery phase, could serve as a new driving force, though the scientific understanding of their potential positive role in the context of mental disorders remains underexplored^10,11^.

Previous models of well-being and mental health have primarily focused on the components of well-being without fully capturing all aspects of mental health as defined by the World Health Organization^12^ and classical theories of mental health. The Maintainable Positive Mental Health Theory (MPMHT)^10^ incorporates all theory-based and empirically identified components of well-being as features of mental health, reflecting the presence and proper functioning of mental health competencies necessary for maintaining and promoting positive psychological status, mental, and physical health—even in the presence of mental disorders. The proposed definition of mental health includes high levels of global well-being, psychological, social, and spiritual functioning, resilience, effective creative and executive functioning, savoring capacities, and coping mechanisms. These pillars ensure that individuals can thrive amid change and challenges, even with symptoms of various mental disorders, declines, losses, and negative life events. The Mental Health Test (MHT)^10,11^ operationalizes the MPMHT and is the first measure offering a comprehensive, five-dimensional framework designed to encompass the wide spectrum of psychological resources related to mental health, including individuals with mental disorders.

Focusing on the benefits of the MPMHT in clinical practice, integrating mental health competencies within psychiatric rehabilitation is essential for delivering effective, individualized, and holistic care. By exploring the positive psychological and behavioral dimensions of individuals with mental disorders, practitioners can establish a robust therapeutic alliance that focuses on setting positive life goals, such as improved psychological functioning, social inclusion, destigmatization, and reduction of self-stigma. This approach enables practitioners to utilize evidence-based practices, promote empowerment, facilitate interdisciplinary collaboration, and address the diverse needs of individuals. These objectives extend beyond mere symptom reduction, aiming to support the fullest possible restoration of daily functioning. A comprehensive assessment of positive psychological attributes in managing mental disorders proposes a new paradigm for rehabilitation, enabling patients to achieve a better quality of life more quickly and to function better even when symptoms persist. This holistic approach not only enhances the subjective experiences of clients, but it has significant economic and societal implications, as more individuals can retain or return to work sooner, and treatment costs can be reduced through faster recovery.

The present study aims to investigate whether the strength of mental health competencies and the severity of mental disorder symptoms, and their interaction, differ in their associations with several dimensions of well-being in Hungarian adult psychiatric and non-clinical samples. Inspired by the study by Cowden and colleagues^13^, our analysis plan first examines whether the strength of mental health competencies and the severity of mental disorder symptoms, and their interaction, differ in their associations with indices of well-being in Hungarian adult psychiatric and non-clinical community samples. We estimate bivariate associations of each with six criterion variables that reflect several dimensions of well-being: emotional well-being, social well-being, spiritual well-being, engagement, meaning, psychological well-being, life satisfaction, and positivity. We expect that mental health competencies, as measured by the MPMHT, will be positively associated with well-being, that symptoms of mental disorders will be negatively associated with well-being, and that effect sizes will vary depending on the strength of mental health competencies and the severity of mental disorder symptoms. We hypothesize that the interaction between mental health competencies and mental disorder symptoms will provide the most accurate prediction of subjective well-being.

Second, we test for further evidence of non-concurrent mental disorder symptoms and mental health competencies by estimating co-occurring categorical combinations of mental disorder symptoms (i.e., low, medium, high) and mental health competencies (i.e., low, medium, high). Consistent with conceptual and empirical literature, we expect to find evidence of low, medium, and high mental health competencies in the presence of low, medium, and high mental disorder symptoms. Specifically, we hypothesize that the highest prevalence will be found in the combination of low mental disorder symptoms and high mental health competencies, and in the combination of high mental disorder symptoms and low mental health competencies. The second highest prevalence is expected in the combination of moderate mental health symptoms and moderate mental disorder symptoms.

## Materials and Methods

The method and analysis plan were preregistered (https://osf.io/s4h8k/). Ethical approval (ethical permission number: IV/2423–3/2022/EKU) was obtained from the national Medical Research Council. All procedures were carried out in accordance with the Declaration of Helsinki on ethical principles for medical research involving human subjects, and with the relevant guidelines and regulations. Written informed consent was obtained from all subjects before participating in the study.

### Study sample

A cross-sectional design was used to measure mental health competencies, mental disorder symptoms, and well-being in psychiatric and non-clinical community samples. In the case of the former sample, data were collected between 22 April 2022 and 2 February 2023 from four Hungarian healthcare facilities under the following conditions. In the Department of Psychiatry and Psychotherapy, Semmelweis University, data were collected from inpatients after their medication had been adjusted (1.5–2 weeks after admission). Outpatients of the Community Psychiatry Centre, Semmelweis University completed the questionnaire at their first medical examination. Data from outpatients at the Psychosomatic Centre of the Institute of Behavioural Sciences, Semmelweis University were collected during the patients’ third therapy session. Data were collected from inpatients at the National Institute of Mental Health, Neurology, and Neurosurgery at the Nyírő Gyula Hospital after medication adjustment (1.5–2 weeks after admission), and from outpatients during their third therapy session. The sample received the information statement, the consent form, and the questionnaire in paper format. In a separate document, the patient’s psychiatrist or clinical psychologist provided information about the diagnosis of the patient’s mental disorder(s), the severity of the presenting symptoms, and the patient’s pharmacotherapy. The inclusion criteria were: (1) age: 18–80 years; (2) voluntary participation; and (3) diagnosis with (a) mental disorder(s). The exclusion criterion was a condition that impaired cognitive function and prevented the completion of the questionnaire. The psychiatric sample consisted of 129 patients (44 male, 85 female), aged M = 39.6 (SD = 14.9).

The non-clinical community sample is a non-representative, convenient sample collected online to provide additional information compared to the psychiatric sample. Participants received the same information statement, consent form and self-report questionnaires. Data were collected between 7 August 2022 and 18 April 2023. A total of 253 Hungarian adults (43 men, 210 women), aged M = 40.1 (SD = 14.1), participated in the study. Further socio-demographic indicators of the samples are presented in Supplementary Table 1.

### Measures

Fourteen questions referred to sociodemographic data. Twenty-seven questions measured general mental state and physical condition and symptoms. One question (Positive experience%) assessed the proportion of the respondent’s recent positive experiences (1 = 10% positive experiences and 90% negative experiences … 9 = 90% positive experiences and 10% negative experiences). Details of the measures are presented below and in Supplementary Tables 2, 3 and 4 and Supplementary Fig. 1.

### Mental health

Participants completed the Mental Health Test (MHT)^10^, which serves as an operationalised, comprehensive measure of the Maintainable Positive Mental Theory^10^. The MHT measures the five pillars of mental health based on psychological competencies and capacities.

The first pillar of the MPMHT is Global Well-being, which integrates existing well-being theories and encompasses multi-component subjective well-being in the emotional, psychological, social, and spiritual domains of life^14–16^. Table 1 outlines the pillars of Global Well-being and their relationship with self-regulation, savoring capacity, resilience, and creative and executive efficiency.

**Table 1:** Pillars of Global Well-being according to Maintainable Positive Mental Health Theory.

Global well-being			
Emotional well-being	Positive functioning		Spiritual well-being
	Psychological well-being	Social well-being	
Global well-being			
Positive affect Happiness Life Satisfaction	Self-acceptance Personal growth Environmental mastery Autonomy Positive relations with others	Social acceptance Social actualization Social contribution Social coherence Social integration	Joy of transcendence experience Joy of universality experience Vertical and horizontal responsibility

The pillars of global well-being refer to the set of mental health characteristics that reflect the presence and proper functioning of the psychological capacities needed to maintain and promote positive psychological status and mental health, according to the Maintainable Positive Mental Health Theory. Table adapted from Ref.^10^.

Savoring is the second pillar and refers to the ability to mentally relive pleasurable memories and experiences, generating mental well-being and extending it to future events^14^. Savoring is a necessary skill for MPMHT, as it contributes to achieving and maintaining positive mental health^17^.

The third pillar is Creative and Executive Efficiency, which enables individuals to cope with difficulties and challenges by mobilising their competencies in individual and social problem solving^14,18^.

The fourth pillar is Self-regulation, which is the ability to regulate and control temperament, emotions, and negative states while persevering to achieve a goal. This ability plays a crucial role in mental health and represents one of the most adaptive variables of human behaviour^19–21^.

Finally, Resilience is the fifth pillar and refers to an individual’s psychological ability to mobilise their resources and maintain positive mental health when faced with unexpected, stressful situations. The higher the level of resilience, the faster an individual can recover from such situations^22–24^.

According to the MPMHT, these five pillars are responsible for an individual’s mental health. The competencies associated with the five pillars can be trained, improved, and strengthened. As such, the pillars provide an easy-to-understand concept for psychiatric and non-clinical populations. First, it provides a structural model for assessing individual capacities and resources (personal sources of resilience, one’s own creativity and executive competencies, and sources of peer support and social connectedness).

Secondly, on the basis of this assessment, people living with mental disorder(s) can establish a balance with their own physical and mental status, as well as with the outside world, by promoting their development, creating a stable state for personal and social functioning (self-regulation) and an equilibrium of positive and negative emotions (coping, savoring). The mindful application of the five-pillar model, and therefore the presence and effective functioning of these elements, can improve mental and physical well-being and social functioning, increase the level of spiritual connectedness, and, through the maintenance/promotion) of mental and physical support and global functioning^25^.

### Mental disorders

Participants completed the Symptom Checklist-90-Revised^26^ from which the General Severity Index was calculated and used to indicate mental disorders symptoms.

### Criterion variables

The measures used were the Global Well-being Scale^14^; the PERMA-Profiler^27^; the Psychological Immune System Inventory, short form (PISI)^18^; the Psychological Well-being Scale^28^; the Satisfaction with Life Scale^29^; the Positivity Scale^30^. Finally, we included in the analysis those variables for which we received a sufficient number of completions in both samples. They are strongly correlated with the other criterion variables see Ref.^10^. In the case of the psychiatric sample, each respondent’s psychiatrist or clinical psychologist was asked to provide a paper report on the patient, including: (1) the name of the patient’s mental disorder(s) according to DSM-5^2^ or ICD^3^ depending on the institution’s protocol; (2) the severity of symptoms; and (3) the patient’s pharmacotherapy.

### Statistical processing

To estimate the connection of mental health competencies and mental disorders symptoms with the selected measures of well-being, we fit a series of ordinary least squares linear regression models^31^. Because we aim to explore and compare the relationship and strength of our main independent variables in a psychiatric and non-clinical setting, all models were fitted separately throughout our analysis for both samples and all well-being related dependent variables.

For both samples and all three dependent variables, we examine four (Table 2) increasingly complex models. M1 is our base model, which only consist of the main demographic and socio-economic control variables, namely age, gender, and educational attainment. M2 introduces the connection of the Mental Health Test’s mean scale with mental health competencies (MHC) as a continuous variable. Compared to M2, M3 introduces the mental disorders symptoms’ (MDS) mean scale also as a continuous variable. Finally, M4 measures whether there is a possible interaction effect between the two main dependent variables. As a supplementary analysis, we also analysed possible interactions between the control variables and the MHC and MDS but did not find any meaningful results (results available from authors).

**Table 2:** Description of the fitted OLS multivariate regression models.

Model	Description
M1	Age + Gender + Education
M2	M1 + MHC
M3	M2 + MDS
M4	M1 + MHC * MDS

To assess the goodness-of-fit of all models, we use four commonly employed measures. First, we compare models using the Bayesian Information Criterion (BIC)^32^ and Akaike Information Criterion (AIC)^33^ of M1–M4, which are measures of relative goodness-of-fit (smaller values indicating a better fit) that penalize the addition of unimportant predictor variables that increase model complexity without improving model performance. Next, we calculate the adjusted coefficient of determination (adjusted R-squared) of all models, where higher values indicate a better fit. Finally, we compare models using a series of ANOVA tests, where a significant (p < 0.05) result indicates that the more complex model fits our data better.

For all models, we test whether the model fulfils the OLS regression assumptions and check for the presence of outlier and/or influential observations which can possibly bias our results^31^. While some observations had to be removed (see removed N in tables) due to being outliers, diagnostic plots and relevant statistics indicate that all presented models are adequate (available from authors). In addition, as the MHC and MDS variables are prone to be correlated, the absence of multicollinearity in models M3 is especially important. We explore the issue using the commonly employed Variance Inflation Factors (VIF, values above 5 indicate potential issues), presented in Supplementary Table 5.

## Results

**Figure 1:**
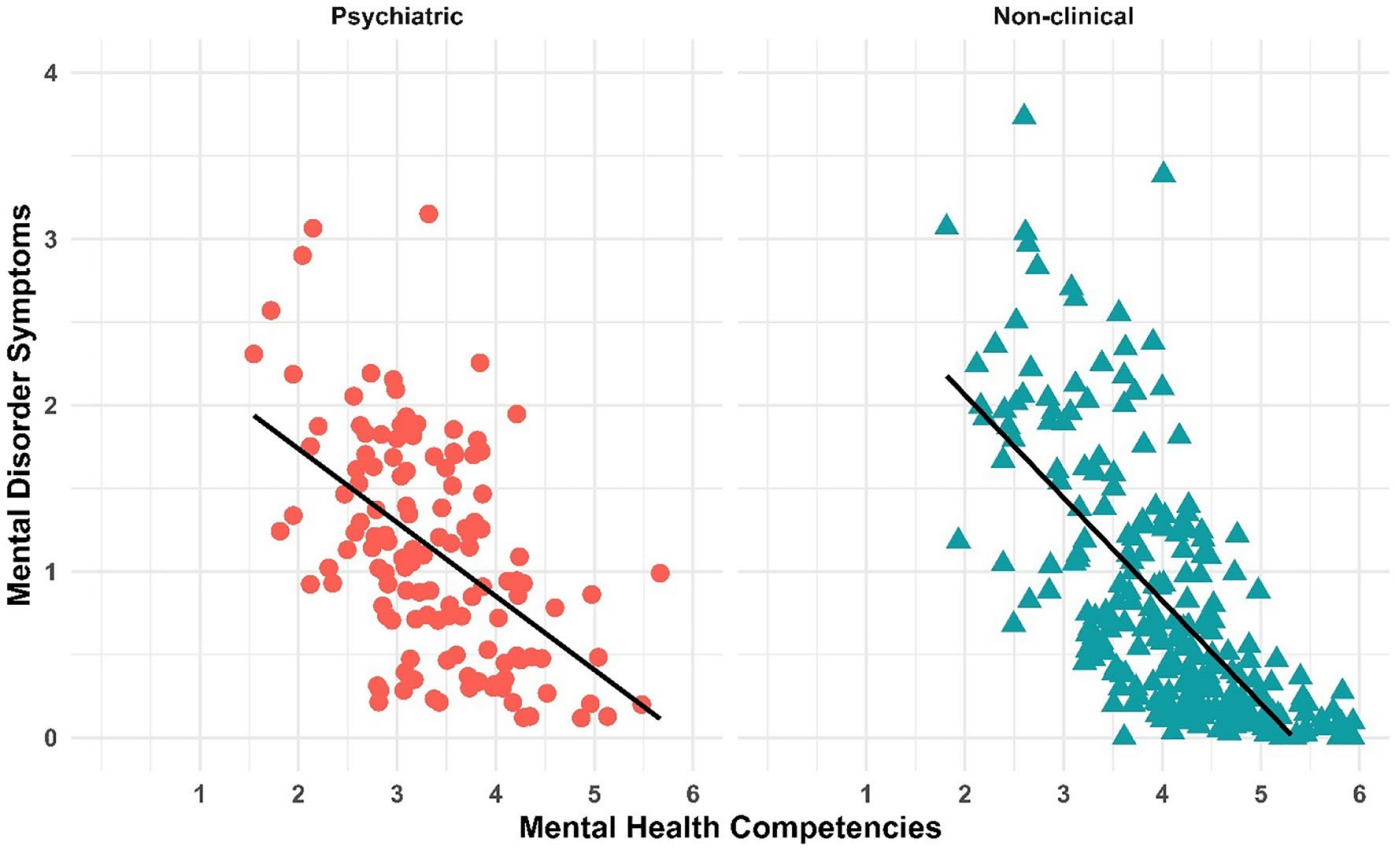
Association between mental health competencies and mental disorders symptoms in both samples. N = 129 for the psychiatric and N = 253 for the non-clinical sample.

The scatterplot on Fig. 1 shows the bivariate relationship between the continuous scales of MHC and MDS for both the psychiatric and the non-clinical sample. Results imply a negative correlation between the two measures for both samples (r =  − 0.52 for the psychiatric and r =  − 0.69 for the non-clinical sample, with *p* < 0.001 in both samples). This implies the evident association between mental health and disorder symptoms: individuals with lower MDS have higher MHC.

As a significant association is present between the two measures, it is not evident whether including both as independent variables in the estimation of well-being improves our models compared to using only one of them. Table 3 contains the model comparisons and fit statistics of the aforementioned models for all three well-being scales and the two samples. For further details, see Supplementary Table 6. Generally, based on the AIC, the BIC, the adjusted R-squared and ANOVA tests, results show that model 3 offers the best fit in all cases. This means that (1) models that include measures of mental health competencies and mental disorder symptoms are better than the models that only include base controls (age, gender and education), and (2) including both the MHC and MDS variables in one model improves model fit – suggesting that they improve our estimations of well-being across the three scales and both samples without substantial multicollinearity (see Supplementary Table 4). Model 4, which proposes an interaction between the MHC and MDS was only found to be the best fitting for the non-clinical sample with the positivity outcome.

**Table 3:** Model comparison and fit statistics.

Model	Psychiatric			Non-clinical		
	Perma	Diener	Positivity	Perma	Diener	Positivity
BIC (Lower better)						
Model 1	453.7	432.9	251.6	958.5	816.8	584.5
Model 2	310.3	365.4	170.2	736.5	576.1	416.5
Model 3	**310**	**359.7**	**170**	**716.8**	**569.7**	411.9
Model 4	314.9	363.6	173.6	722.3	572.9	**408.9**
AIC (Lower better)						
Model 1	437.3	416.2	235.2	937.5	795.9	563.4
Model 2	291.1	345.8	151.1	712	551.6	391.9
Model 3	**289**	**337.3**	**148.3**	**688.8**	**541.7**	383.9
Model 4	290.3	338.4	149.1	690.8	542	**377.4**
Adjusted R-squared (Higher better)						
Model 1	0.04	0.07	0.07	0	0.04	0.02
Model 2	0.73	0.48	0.56	0.60	0.65	0.51
Model 3	**0.74**	**0.52**	**0.58**	**0.64**	**0.67**	0.53
Model 4	0.74	0.52	0.57	0.64	0.63	**0.55**
Model comparison using ANOVA (tests better model fit)						
Model 1	–	–	–	–	–	–
Model 2	293.9***	101.8***	25.44***	247.8***	433.9***	262.9***
Model 3	4.0*	10.3**	0.94*	25.8***	6.18***	64.3**
Model 4	0.73	0.35	0.22	0.02	2.08	62.1**
N	115	121	113	244	244	245
Removed N	3	3	5	4	5	4

Significant tests (*p* < 0.05) indicate better fit in the ANOVA. Model 2 test compared Model 2 to Model 1, Model 3 tests compare Model 3 to Model 2, and Model 4 tests compare Model 4 to Model 3. Removed N indicates the number of outlier/highly influential observations omitted from the models.

Significant values are in bold.

**Figure 2:**
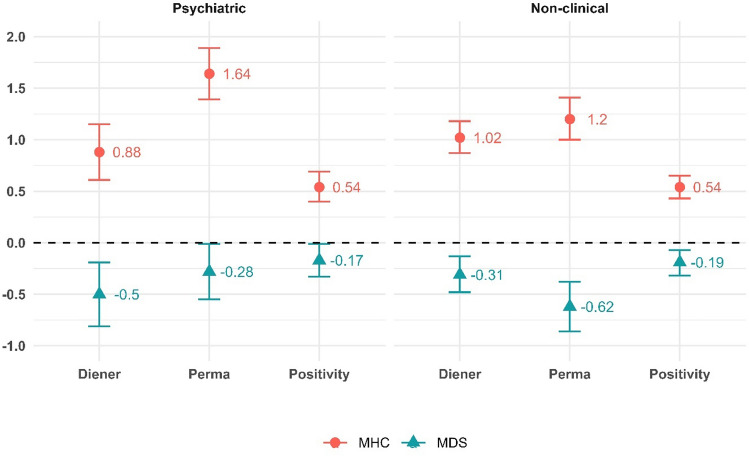
Main effect (unstandardized betas) of the MHC and MDS variables from model 3. N = 113–121 for the clinical and N = 244–245 for the non-clinical sample. All effects are significant (*p* < 0.05). The dotted line indicates zero effect. The bars indicate the 95% confidence interval.

As the discussed fit statistics and model comparisons already suggested, the MHC and MDS of the individuals affect their well-being in the presence of base controls. This relationship (all being statistically significant at *p* < 0.05) is presented on Fig. 2, with the 95% confidence intervals indicated by the error bars (detailed models available in Supplementary material). Generally, results are as expected. In both a psychiatric and non-clinical setting, a 1 scale-point increase in MHC improved the estimated level of well-being. The unstandardized betas indicate that the effect sizes are similar across samples for the Positivity outcome but are slightly weaker (0.88 compared to 1.02) for the Diener, and stronger (1.64 compared to 1.2 in the non-clinical sample) for the PERMA outcomes in the psychiatric sample. For the MDS, all effects indicate that a 1 scale-point increase in MDS decreased well-being across all models and samples. Again, relationships are similar for the Positivity scale, but MDS is more negative in the case of the Diener and less negative in the case of the PERMA scale for the psychiatric sample.

**Figure 3:**
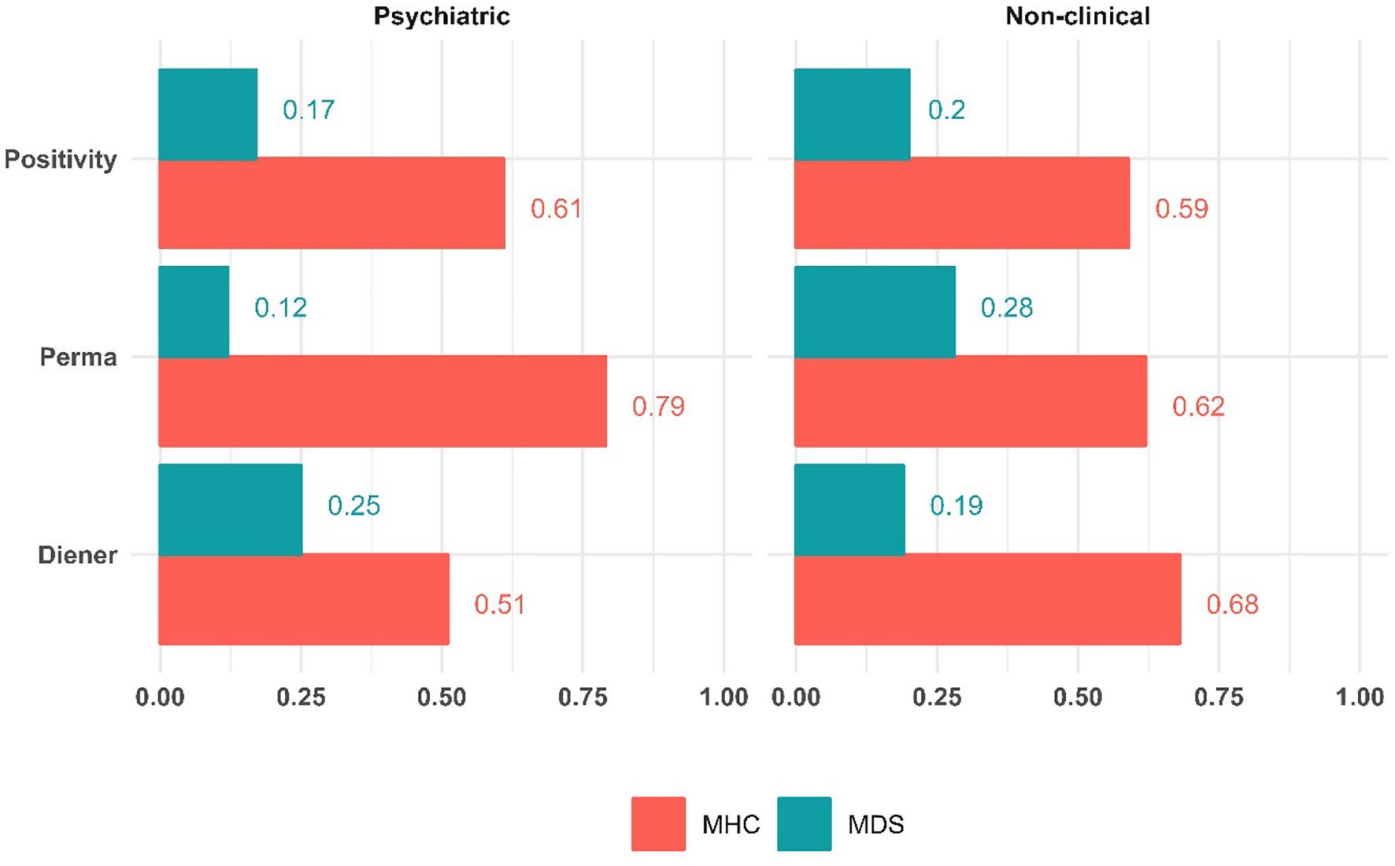
Absolute values of the standardized effect of the MHC and MDS variables from M3. N = 113–121 for the clinical and N = 244–245 for the non-clinical sample. All effects are significant (*p* < 0.05).

To evaluate whether the MHC and MDS has the stronger connection with well-being, we also present the standardized betas of the models on Fig. 3. Note that for ease of comparison, we took the absolute values of the MHC and MDS standardized effects. In all models and both samples (and invariant to the order of inclusion into the models), the MHC were found to be stronger determinants of well-being than the MDS.

**Figure 4:**
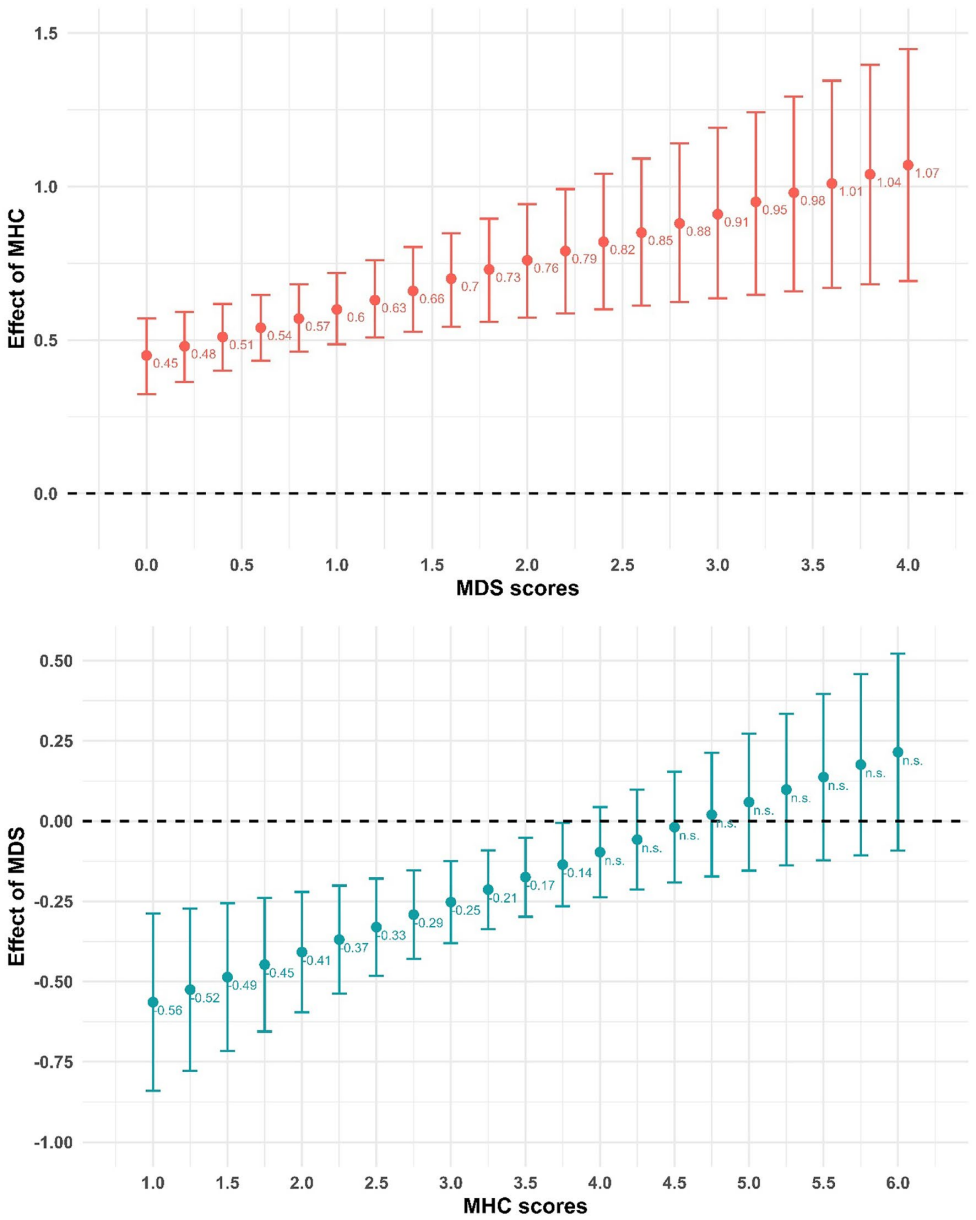
The conditional effect of MHC (upper) and MDS (lower) in the non-clinical sample estimating Positivity Scale. N = 245. All effects are significant (*p* < 0.05), unless otherwise noted with (n.s.). The dotted line indicates zero effect. The bars indicate the 95% confidence interval.

Lastly in our analysis, we explored the significant interaction effect between MHC and MDS for the positivity outcome in the non-clinical sample, which was shown to be the best fitting model according to the fit statistics and model comparisons in Table 2 (the detailed model is available in Supplementary Table 7). The interaction term indicates that the effect of MHC (MDS) is conditional on the value of MDS (MHC). To better understand this conditionality, Fig. 4 present the unstandardized coefficients of the given scale conditional on the values of the other one.

Regarding the conditional effect of MHC, it can be said that a 1 scale-point increase in mental health competencies have an increasingly stronger connection (from 0.45 at MDS = 0 to 1.07 at MDS = 4) with positivity in the non-clinical sample. To put it simply, this indicates that in the presence of more severe mental disorder symptoms, mental health competencies are stronger determinants of estimated well-being. Conversely, the negative influence of MDS weakens as MHC increase (− 0.56 to non-significance). This implies that with higher mental health competencies, well-being is estimated to decrease less in the presence of mental health disorder symptoms and importantly, with very higher MHC scores (4.0 or above on the scale), the presence of these symptoms does not affect well-being at all.

To illustrate the prevalence of certain MHC and MDS combinations implied by the above-presented interaction effect, Table 4 contains the crosstabulation along the terciles of the MHT and the SCL-90-R in the non-clinical sample, respectively. While (expectedly) most observations show that changing MHC levels are associated with expected changes in MDS (lower MHC goes with higher MDS and vice versa), other hypothesised combinations are present as well. For example, 10.6% of all observations have low MHC with medium MDS, and 11.0% have medium MHC and high MDS. Although this categorization can be considered crude, it still illustrates well that the interaction revealed above is a worthwhile contribution and not just a theoretical occurrence.

**Table 4:** Crosstabulation of MHC and MDS categories in the non-clinical sample estimating positivity.

MHC/MDS	Low (0–0.24)	Medium (0.24–0.83)	High (0.83–4)
Low (0–3.7)	3 (1.2%)	26 (10.6%)	52 (21.2%)
Medium (3.7–4.4)	24 (9.8%)	30 (12.2%)	27 (11.0%)
High (4.4–6)	54 (22.0%)	24 (9.8%)	5 (2.0%)

N = 245 for the non-clinical sample, estimating positivity. Parentheses contain total percentages of a given category combination.

## Discussion

The aim of the present study was to examine the impact of mental health competencies and symptoms of mental disorders on subjective well-being in Hungarian adult psychiatric and non-clinical community samples. The findings of high practical relevance are summarised in six points.

1. Our study shows strong significant negative association between mental health competencies and mental disorder symptoms for both samples (r =  − 0.52 for the psychiatric and r =  − 0.69 for the non-clinical sample, with *p* < 0.001 in both samples). This means that people with stronger mental health competencies have weaker mental disorder symptoms and vice versa. The results are consistent with previous findings see e.g. Ref.^34^ , but this is the first study to examine mental health competencies in relation to well-being and mental disorders.
2. Regression models in both samples that include measures of mental health competencies and mental disorder symptoms predict subjective well-being better than the models that include only demographic indicators (age, sex, and education). Furthermore, including both the mental health competencies and mental disorder symptoms variables in one model in both samples can predict patients’ well-being even more accurately. The practical significance of this is that if we want to estimate a patient’s subjective well-being, we get a more accurate picture if we also take into account the existing mental health competencies in addition to mental disorder symptoms.
3. As we hypothesised, mental health competencies were positively related to subjective well-being, mental disorder symptoms were negatively related to subjective well-being, and there were some differences in effect sizes depending on the strength of mental health competencies and the severity of mental disorder symptoms.
4. We tested whether mental health competencies and mental disorder symptoms had the stronger relationship with well-being. In all models and both samples, the mental health competencies were found to be stronger determinants of well-being than mental disorder symptoms. This provides empirical support that exploring the positive dimensions of people living with mental disorders can be a solid basis for achieving positive life goals, where the goal is not only symptom reduction, but the restoration of everyday functioning to the greatest extent possible.
5. Another important finding is that the interaction of mental health competencies and mental disorders is no more predictive of well-being in either clinical or non-clinical samples than when the effects of each are considered separately. The lack of interaction can be seen as a positive finding, as mental health competencies are stronger predictors of well-being on their own. This suggests that focusing on mental health competencies is as important, if not more important, than the symptom reduction in the process of rehabilitation practice to increase subjective well-being.
6. An exception is the significant interaction in the case of the Positivity scale in the community sample. In the presence of more disorder symptoms, mental health competencies are stronger determinants of positive orientation. The negative influence of mental disorder symptoms decreases with increasing mental health competencies (-0.56 to non-significance). This means that at higher levels of mental health competencies, well-being is estimated to decrease less in the presence of mental health disorder symptoms. Importantly, at very high scores of mental health competencies (4.0 or above on the scale), the presence of mental disorder symptoms do not affect positivity at all. Analyses using categorical combinations of depression and distress showed that changing levels of mental health competencies are associated with expected changes in mental disorder symptoms (lower level of mental health competencies are associated with higher mental disorder symptoms and vice versa). It is therefore worthwhile to invest energy in the support and development of mental health competencies, which can increase positivity and minimise the negative effects of mental disorders in non-clinical samples.

This study is not without limitations. Firstly, the analyses were carried out on a convenience community sample and the sample therefore is not representative. Secondly, self-report questionnaires are to some extent subject to the conscious and unconscious biases of respondents. In addition, because participation was voluntary, more severe cases were not represented, while various factors related to personality, illness, and attitudes, as well as factors related to mental well-being, are likely to have influenced willingness to participate. Future studies should perform the above analyses in separate patient or age groups and according to the five pillars of the MPMHT, filtering out the effect of medication, psychotherapy, and the type of health care.

## Conclusion

The present study proposes a model for investigating the complex interrelationship between mental disorders, mental health competencies and well-being. Mental health competencies, as conceptualised in the Maintainable Positive Mental Health Theory^10^ and included in the Mental Health Test^10,11^, have an important predictive value for well-being in the presence of psychopathological symptoms and/or mental disorders. In addition to their predictive value, mental health competencies are useful in other ways. The implementation of appropriate psychological interventions aimed at supporting, strengthening, and developing mental health competencies can be effectively employed in therapeutic and rehabilitative programmes. Furthermore, in the absence of mental disorders, these competencies can serve as a robust foundation for mental health promotion. Further research should be conducted to identify additional strategies. Furthermore, the wider use of mental health competencies enhancement strategies in conjunction with appropriate treatment in intervention programmes could be a significant factor in the treatment of mental disorders. With the recognition of the importance of symptom reduction, the enhancement of mental health competencies may become the main focus in the individual conceptualisation and treatment plan of a patient with mental disorders.

### Supplementary Information

Supplementary Information.

## Data Availability

The datasets generated and/or analysed during the present study are available from the corresponding author on reasonable request.
