# Supplementary material for: Mental health competencies are stronger determinants of well-being than mental disorder symptoms in both psychiatric and non-clinical samples
Source: Sci Rep. 2024 Jun 5;14:12943. doi: 10.1038/s41598-024-63674-9 (PMC11153550; doi:10.1038/s41598-024-63674-9)
Supplement: Supplementary file 1 — Supplementary Information. [file 41598_2024_63674_MOESM1_ESM.docx]

**Table 1. Sociodemographic indicators of the clinical and non-clinical community sample**

|  | Psychiatric | Non-clinical |
| --- | --- | --- |
| Age | 39.6 (14.9) | 40.1 (14.1) |
| Gender | | |
| Male | 34.1% | 16.9% |
| Female | 65.9% | 83.1% |
| Education | | |
| Elementary or lower | 5.4% | 1.9% |
| Secondary | 49.6% | 31.2% |
| Tertiary | 45.0% | 66.8% |
| MHC | 3.37 (0.78) | 4.07 (0.85) |
| MDS | 1.13 (0.67) | 0.78 (0.76) |
| N | **129** | **253** |

**Table 2. Details of measures**

| **Name of the measure** | **Description of the measure** |
| --- | --- |
| Mental Health Test [10, 11] | The 17-item self-report test operationalises the Maintainable Positive Mental Health Theory^13,14^ and measures five mental health capacities and competencies in adult population: Global Well-being, Savoring, Resilience, Self-regulation, and Creative and Executive Efficiency. Respondents rate 17 items on a 7-point Likert scale from 1 (strongly disagree) to 7 (strongly agree). The MHT score is defined as the average of the scores for the five subscales, which provide a comprehensive picture of the respondent’s level of mental health capacities. |
| Global Well-being Scale [14] | This self-report questionnaire operationalises the bio-psycho-socio-spiritual model of well-being, which emphasises that the condition for complete well-being is to function well in the emotional, psychological, social and spiritual domains of one's human nature. The 17 statements in the questionnaire are rated on a 7-point Likert scale from 1 (strongly disagree) to 7 (strongly agree). |
| PERMA-Profiler [27, 35] | The five-pillar model measures positive emotions, engagement, positive relationship, meaning, accomplishment which reinforce one another in creating and maintaining a state of well-being. The self-report 23-items are rated on a 10-point Likert scale from 1 (strongly disagree) to 10 (strongly agree). |
| Psychological Well-being Scale [28] | This widely used self-report scale operationalises an improved version of Diener's concept of subjective well-being, which emphasises the need for competence, optimism, contribution to the well-being of others, purpose in life, self-esteem, and positive relationships, in addition to life satisfaction and the dominance of positive emotions. The 8 statements in the questionnaire are rated on a 7-point Likert scale from 1 (strongly disagree) to 7 (strongly agree). There is currently no Hungarian adaptation of the scale available, only an official translation by Judit Görgényi. Several studies have demonstrated the applicability of the Hungarian version (e.g. Ref [27, 36]). |
| Satisfaction with Life Scale [29, 37] | This self-report scale measures overall life satisfaction by rating 5 items on a seven-point Likert scale from 1 (strongly disagree) to 7 (strongly agree). |
| Positivity Scale [30] | Respondents rate on a self-report basis the 8 items on a 5-point scale from 1 (strongly disagree) to 5 (strongly agree) that measure the individual's tendency to view their life and experiences in a positive way. |
| Symptom Checklist-90, revised [26, 38] | This self-report inventory measures the presence and degree of severity of psychological symptoms on 9 scales: somatization, compulsivity, interpersonal sensitivity, depression, anxiety, hostility, phobia, paranoia, psychoticism, and a group of additional items not included in any of the scales. The Global Severity Index, in contrast to the problem-specific scales, provides an overall picture of the general severity of mental disorder symptoms. The 90 statements in the questionnaire are rated on a 5-point Likert scale. |

**Table 3. Reliability measures for the main variables**

| Variable | Psychiatric | Non-clinical |
| --- | --- | --- |
| Cronbach’s α | | |
| MHC | 0.74 | 0.78 |
| MDS | 0.93 | 0.97 |
| Perma | 0.87 | 0.89 |
| Diener | 0.90 | 0.91 |
| Positivity | 0.78 | 0.83 |
| Variable | **Psychiatric** | **Non-clinical** |
| McDonald’s ω | | |
| MHC | 0.77 | 0.79 |
| MDS | 0.93 | 0.97 |
| Perma | 0.88 | 0.89 |
| Diener | 0.91 | 0.91 |
| Positivity | 0.82 | 0.86 |

**Table 4. Correlation of subscales for the main variables**

**MHC**

| *Psychiatric* | Global Well-Being | Savoring | Creative and Executive Efficiency | Self-regulation | Resilience |
| --- | --- | --- | --- | --- | --- |
| Global Well-Being | 1 |  |  |  |  |
| Savoring | 0.58 ** | 1 |  |  |  |
| Creative and Executive Efficiency | 0.48 ** | 0.51 ** | 1 |  |  |
| Self-regulation | 0.21 ** | -0.02 | -0.03 | 1 |  |
| Resilience | 0.67 ** | 0.48 ** | 0.48 ** | 0.31 ** | 1 |
| *Non-clinical* | **Global Well-Being** | **Savoring** | **Creative and Executive Efficiency** | **Self-regulation** | **Resilience** |
| Global Well-Being | 1 |  |  |  |  |
| Savoring | 0.63 ** | 1 |  |  |  |
| Creative and Executive Efficiency | 0.46 ** | 0.52 ** | 1 |  |  |
| Self-regulation | 0.31 ** | 0.13 * | 0.10 | 1 |  |
| Resilience | 0.62 ** | 0.54 ** | 0.52 ** | 0.35 ** | 1 |

*** p < 0.001; ** p < 0.01, * p < 0.05. Cell values are Pearson correlation coefficients.

**MDS**

| *Psychiatric* | SOM | OC | IS | DEP | ANX | HOS | PHOB | PAR | PS |
| --- | --- | --- | --- | --- | --- | --- | --- | --- | --- |
| SOM | 1 |  |  |  |  |  |  |  |  |
| OC | 0,37 ** | 1 |  |  |  |  |  |  |  |
| IS | 0,45 ** | 0,71 ** | 1 |  |  |  |  |  |  |
| DEP | 0,56 ** | 0,72 ** | 0,73 ** | 1 |  |  |  |  |  |
| ANX | 0,72 ** | 0,68 ** | 0,70 ** | 0,78 ** | 1 |  |  |  |  |
| HOS | 0,44 ** | 0,46 ** | 0,53 ** | 0,49 ** | 0,60 ** | 1 |  |  |  |
| PHOB | 0,46 ** | 0,50 ** | 0,57 ** | 0,43 ** | 0,67 ** | 0,46 ** | 1 |  |  |
| PAR | 0,47 ** | 0,60 ** | 0,79 ** | 0,60 ** | 0,67 ** | 0,61 ** | 0,57 ** | 1 |  |
| PS | 0,43 ** | 0,74 ** | 0,72 ** | 0,63 ** | 0,62 ** | 0,56 ** | 0,41 ** | 0,75 ** | 1 |
| *Non-clinical* | **SOM** | **OC** | **IS** | **DEP** | **ANX** | **HOS** | **PHOB** | **PAR** | **PS** |
| SOM | 1 |  |  |  |  |  |  |  |  |
| OC | 0.75 ** | 1 |  |  |  |  |  |  |  |
| IS | 0.70 ** | 0.78 ** | 1 |  |  |  |  |  |  |
| DEP | 0.76 ** | 0.91 ** | 0.82 ** | 1 |  |  |  |  |  |
| ANX | 0.82 ** | 0.86 ** | 0.82 ** | 0.89 ** | 1 |  |  |  |  |
| HOS | 0.69 ** | 0.73 ** | 0.74 ** | 0.74 ** | 0.77 ** | 1 |  |  |  |
| PHOB | 0.75 ** | 0.71 ** | 0.70 ** | 0.71 ** | 0.79 ** | 0.63 ** | 1 |  |  |
| PAR | 0.73 ** | 0.76 ** | 0.83 ** | 0.77 ** | 0.79 ** | 0.74 ** | 0.69 ** | 1 |  |
| PS | 0.78 ** | 0.81 ** | 0.78 ** | 0.80 ** | 0.84 ** | 0.73 ** | 0.74 ** | 0.76 ** | 1 |

*** p < 0.001; ** p < 0.01, * p < 0.05. Cell values are Pearson correlation coefficients.

**Perma**

| *Psychiatric* | POSEM | ENGAG | POZREL | MEANING | ACCOMP | NEGEM | HEALTH |
| --- | --- | --- | --- | --- | --- | --- | --- |
| POSEM | 1 |  |  |  |  |  |  |
| ENGAG | 0.53 ** | 1 |  |  |  |  |  |
| POZREL | 0.62 ** | 0.36 ** | 1 |  |  |  |  |
| MEANING | 0.77 ** | 0.57 ** | 0.61 ** | 1 |  |  |  |
| ACCOMP | 0.74 ** | 0.60 ** | 0.43 ** | 0.74 ** | 1 |  |  |
| NEGEM | 0.46 ** | 0.12 | 0.24 ** | 0.32 ** | 0.22 * | 1 |  |
| HEALTH | 0.62 ** | 0.41 ** | 0.43 ** | 0.61 ** | 0.51 ** | 0.28 ** | 1 |
| *Non-clinical* | **POSEM** | **ENGAG** | **POZREL** | **MEANING** | **ACCOMP** | **NEGEM** | **HEALTH** |
| POSEM | 1 |  |  |  |  |  |  |
| ENGAG | 0.67 ** | 1 |  |  |  |  |  |
| POZREL | 0.77 ** | 0.52 ** | 1 |  |  |  |  |
| MEANING | 0.83 ** | 0.75 ** | 0.73 ** | 1 |  |  |  |
| ACCOMP | 0.70 ** | 0.64 ** | 0.51 ** | 0.78 ** | 1 |  |  |
| NEGEM | 0.43 ** | 0.08 | 0.27 ** | 0.19 ** | 0.16 * | 1 |  |
| HEALTH | 0.68 ** | 0.48 ** | 0.53 ** | 0.64 ** | 0.65 ** | 0.23 ** | 1 |

*** p < 0.001; ** p < 0.01, * p < 0.05. Cell values are Pearson correlation coefficients. Abbrevations: (POSEM) = Positive Emotions, (ENGAG) = Engagement, (POZREL) = Positive Relationships, (MEANING) = Meaning, (ACCOMP) = Accomplishment, (NEGEM) = Negative Emotions, (HEALTH) = Health.

**Diener**

| *Psychiatric* | D1 | D2 | D3 | D4 | D5 | D6 | D7 | D8 |
| --- | --- | --- | --- | --- | --- | --- | --- | --- |
| D1 | 1 |  |  |  |  |  |  |  |
| D2 | 0.56 ** | 1 |  |  |  |  |  |  |
| D3 | 0.66 ** | 0.47 ** | 1 |  |  |  |  |  |
| D4 | 0.56 ** | 0.47 ** | 0.62 ** | 1 |  |  |  |  |
| D5 | 0.49 ** | 0.30 ** | 0.42 ** | 0.67 ** | 1 |  |  |  |
| D6 | 0.67 ** | 0.57 ** | 0.52 ** | 0.61 ** | 0.47 ** | 1 |  |  |
| D7 | 0.67 ** | 0.44 ** | 0.51 ** | 0.47 ** | 0.53 ** | 0.64 ** | 1 |  |
| D8 | 0.65 ** | 0.44 ** | 0.40 ** | 0.58 ** | 0.49 ** | 0.65 ** | 0.56 ** | 1 |
| *Non-clinical* | **D1** | **D2** | **D3** | **D4** | **D5** | **D6** | **D7** | **D8** |
| D1 | 1 |  |  |  |  |  |  |  |
| D2 | 0.57 ** | 1 |  |  |  |  |  |  |
| D3 | 0.71 ** | 0.54 ** | 1 |  |  |  |  |  |
| D4 | 0.47 ** | 0.39 ** | 0.47 ** | 1 |  |  |  |  |
| D5 | 0.52 ** | 0.31 ** | 0.57 ** | 0.56 ** | 1 |  |  |  |
| D6 | 0.70 ** | 0.54 ** | 0.66 ** | 0.53 ** | 0.57 ** | 1 |  |  |
| D7 | 0.67 ** | 0.52 ** | 0.71 ** | 0.46 ** | 0.47 ** | 0.73 ** | 1 |  |
| D8 | 0.52 ** | 0.47 ** | 0.57 ** | 0.48 ** | 0.50 ** | 0.67 ** | 0.59 ** | 1 |

*** p < 0.001; ** p < 0.01, * p < 0.05. Cell values are Pearson correlation coefficients.

**Positivity**

| *Psychiatric* | P1 | P2 | P3 | P4 | P5 | P6 | P7 | P8 |
| --- | --- | --- | --- | --- | --- | --- | --- | --- |
| P1 | 1 |  |  |  |  |  |  |  |
| P2 | 0.50 ** | 1 |  |  |  |  |  |  |
| P3 | 0.41 ** | 0.44 ** | 1 |  |  |  |  |  |
| P4 | 0.70 ** | 0.64 ** | 0.50 ** | 1 |  |  |  |  |
| P5 | 0.50 ** | 0.75 ** | 0.35 ** | 0.63 ** | 1 |  |  |  |
| P6 | -0.16 | -0.21 * | -0.01 | -0.14 | -0.18 | 1 |  |  |
| P7 | 0.34 ** | 0.43 ** | 0.21* | 0.44 ** | 0.52 ** | -0.14 | 1 |  |
| P8 | 0.34 ** | 0.48 ** | 0.13 | 0.33 ** | 0.51 ** | -0.27 ** | 0.54 ** | 1 |
| *Non-clinical* | **P1** | **P2** | **P3** | **P4** | **P5** | **P6** | **P7** | **P8** |
| P1 | 1 |  |  |  |  |  |  |  |
| P2 | 0.70 ** | 1 |  |  |  |  |  |  |
| P3 | 0.39 ** | 0.55 ** | 1 |  |  |  |  |  |
| P4 | 0.82 ** | 0.69 ** | 0.47 ** | 1 |  |  |  |  |
| P5 | 0.64 ** | 0.80 ** | 0.45 ** | 0.65 ** | 1 |  |  |  |
| P6 | -0.23 ** | -0.26 ** | 0.00 | -0.26 ** | -0.18 ** | 1 |  |  |
| P7 | 0.55 ** | 0.65 ** | 0.42 ** | 0.57 ** | 0.65 ** | -0.13* | 1 |  |
| P8 | 0.49 ** | 0.59 ** | 0.31 ** | 0.50 ** | 0.60 ** | -0.22 ** | 0.69 ** | 1 |

*** p < 0.001; ** p < 0.01, * p < 0.05. Cell values are Pearson correlation coefficients.

**Table 5. Variance Inflation Factor and Tolerance for the models used for estimations**

| **VIF** |  | | **Psychiatric** | |  | |  | | **Non-clinical** | |  | |  |
| --- | --- | --- | --- | --- | --- | --- | --- | --- | --- | --- | --- | --- | --- |
|  | **Perma** | **Diener** | | **Positivity** | | **Perma** | | **Diener** | | **Positivity**  **(M3)** | | **Positivity**  **(M4)** |  |
| Age | 1.08 | 1.04 | | 1.02 | | 1.29 | | 1.26 | | 1.25 | | 1.26 |  |
| Gender | 1.02 | 1.02 | | 1.02 | | 1.01 | | 1.01 | | 1.01 | | 1.03 |  |
| Education | 1.19 | 1.13 | | 1.13 | | 1.20 | | 1.21 | | 1.16 | | 1.17 |  |
| MHC | 1.58 | 1.56 | | 1.69 | | 1.97 | | 2.00 | | 1.94 | | 1.23 |  |
| MDS | 1.50 | 1.52 | | 1.62 | | 2.12 | | 2.13 | | 2.11 | | 1.23 |  |
| **Tolerance** | |  | | **Psychiatric** | |  | |  | | **Non-clinical** | |  | |
|  |  | **Perma** | **Diener** | | **Positivity** | | **Perma** | | **Diener** | | **Positivity**  **(M3)** | | **Positivity**  **(M4)** |
| Age | | 0.92 | 0.96 | | 0.98 | | 0.77 | | 0.79 | | 0.79 | | 0.79 |
| Gender | | 0.98 | 0.98 | | 0.98 | | 0.99 | | 0.98 | | 0.99 | | 0.98 |
| Education | | 0.84 | 0.89 | | 0.89 | | 0.83 | | 0.83 | | 0.86 | | 0.86 |
| MHC | | 0.63 | 0.64 | | 0.59 | | 0.51 | | 0.49 | | 0.51 | | 0.81 |
| MDS | | 0.66 | 0.66 | | 0.62 | | 0.47 | | 0.47 | | 0.47 | | 0.81 |

**Table 6. Model 3 of well-being outcomes**

| Variable | Psychiatric | | | | | | | | |
| --- | --- | --- | --- | --- | --- | --- | --- | --- | --- |
|  | **Perma** | | | **Diener** | | | **Positivity** | | |
|  | **Beta** | **S.E.** | **P-value** | **Beta** | **S.E.** | **P-value** | **Beta** | **S.E.** | **P-value** |
| Intercept | 1.59 | 0.66 | **0.017** | 1.81 | 0.68 | **0.008** | 1.4 | 0.38 | **<0.001** |
| Age | -0.01 | 0.006 | **0.024** | -0.02 | 0.006 | **<0.001** | -0.01 | 0.003 | **0.006** |
| Female (ref.: Male) | 0.237 | 0.166 | 0.154 | -0.03 | 0.18 | 0.868 | 0.01 | 0.09 | 0.849 |
| Education  (ref.: Elementary or lower) |  |  |  |  |  |  |  |  |  |
| Secondary | -0.88 | 0.51 | 0.086 | 1.01 | 0.45 | **0.029** | 0.49 | 0.28 | 0.073 |
| Tertiary | -0.89 | 0.52 | 0.086 | 0.92 | 0.46 | 0.049 | 0.49 | 0.28 | 0.078 |
| MHC | 1.64 | 0.13 | **<0.001** | 0.88 | 0.14 | **<0.001** | 0.54 | 0.07 | **<0.001** |
| MDS | -0.28 | 0.13 | **0.049** | -0.50 | 0.16 | **0.002** | -0.17 | 0.08 | **0.033** |
| Variable | **Non-clinical** | | | | | | | | |
|  | **Perma** | | | **Diener** | | | **Positivity** | | |
|  | **Beta** | **S.E.** | **P-value** | **Beta** | **S.E.** | **P-value** | **Beta** | **S.E.** | **P-value** |
| Intercept | 1.89 | 0.89 | **0.045** | 0.68 | 0.75 | 0.363 | 1.67 | 0.39 | **<0.001** |
| Age | -0.02 | 0.005 | **0.002** | 0.001 | 0.004 | 0.897 | -0.003 | 0.002 | 0.195 |
| Female (ref.: Male) | 0.35 | 0.17 | **0.039** | 0.35 | 0.124 | **0.006** | 0.26 | 0.09 | **0.004** |
| Education  (ref.: Elementary or lower) |  |  |  |  |  |  |  |  |  |
| Secondary | 0.91 | 0.59 | 0.127 | 0.36 | 0.54 | 0.501 | -0.07 | 0.24 | 0.789 |
| Tertiary | 0.72 | 0.58 | 0.214 | 0.34 | 0.53 | 0.512 | -0.14 | 0.24 | 0.564 |
| MHC | 1.2 | 0.10 | **<0.001** | 1.02 | 0.08 | **<0.001** | 0.54 | 0.06 | **<0.001** |
| MDS | -0.62 | 0.12 | **<0.001** | -0.31 | 0.09 | **0.001** | -0.19 | 0.06 | **0.002** |

**Table 7. Positivity Model 4 for the non-clinical sample with interaction term**

| Variable | Psychiatric | | |
| --- | --- | --- | --- |
|  | **Perma** | | |
|  | **Beta** | **S.E.** | **P-value** |
| Intercept | 2.07 | 0.41 | **<0.001** |
| Age | -0.003 | 0.003 | 0.293 |
| Female (ref.: Male) | 0.23 | 0.09 | **0.011** |
| Education (ref.: Elementary or lower) |  |  |  |
| Secondary | -0.13 | 0.24 | 0.602 |
| Tertiary | -0.19 | 0.24 | 0.408 |
| MHC | 0.45 | 0.06 | **<0.001** |
| MDS | -0.72 | 0.19 | **<0.001** |
| MHC * MDS | 0.16 | 0.05 | **0.004** |

**Figure 1. Residual diagnostics for presented models (Model 3 and interaction model)**

***Psychiatric sample***

*Perma*

*
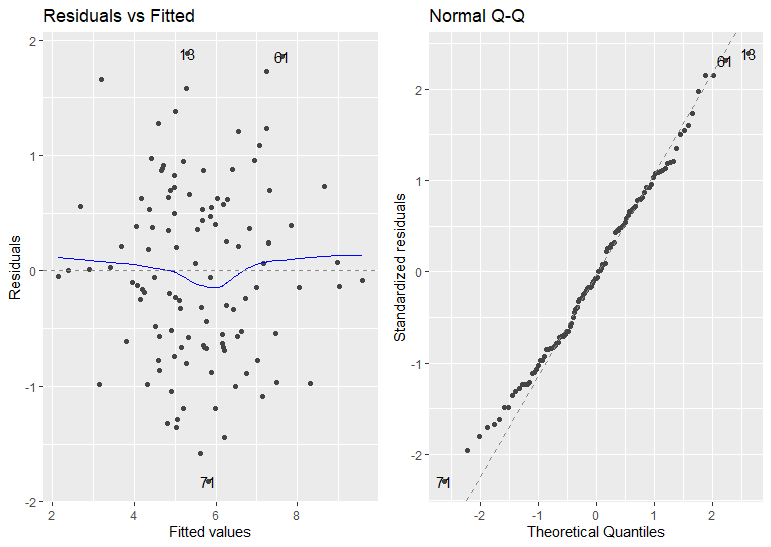
*

*Diener*

*
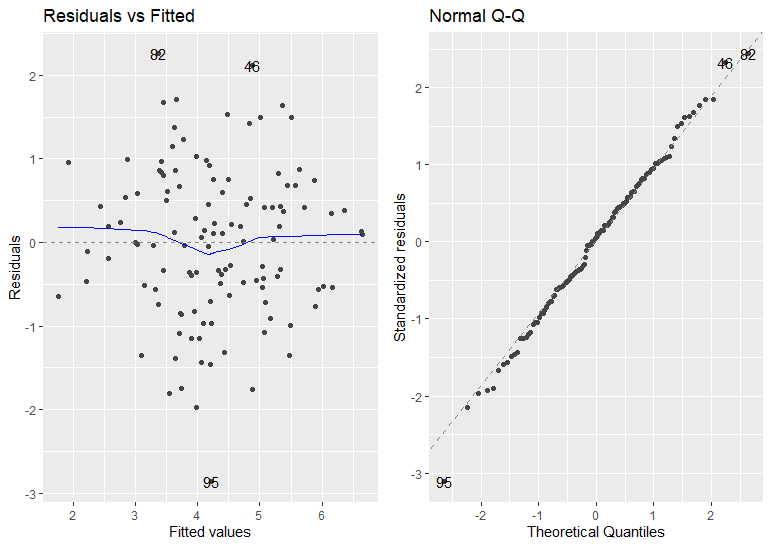
*

*Positivity*

*
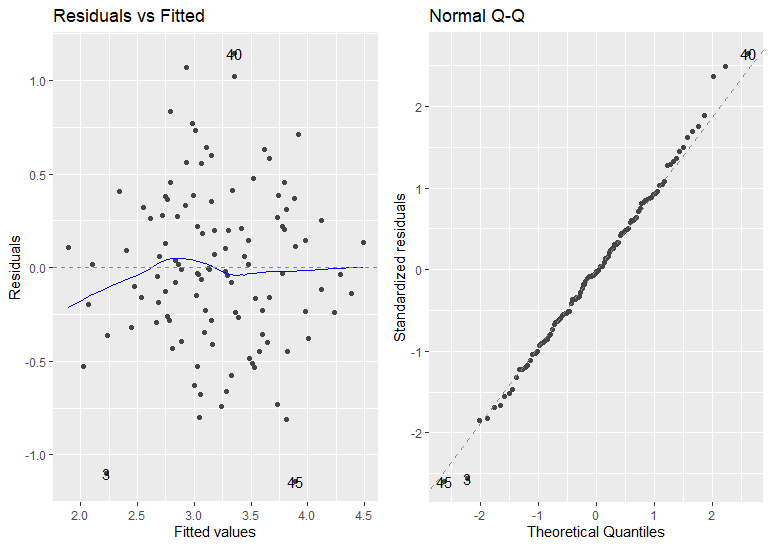
*

***Non-clinical sample***

*Perma*

*
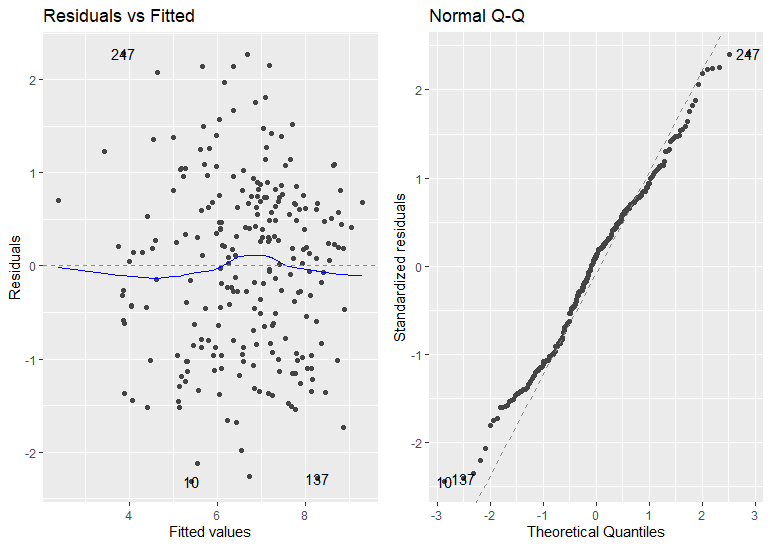
*

*Diener*

*
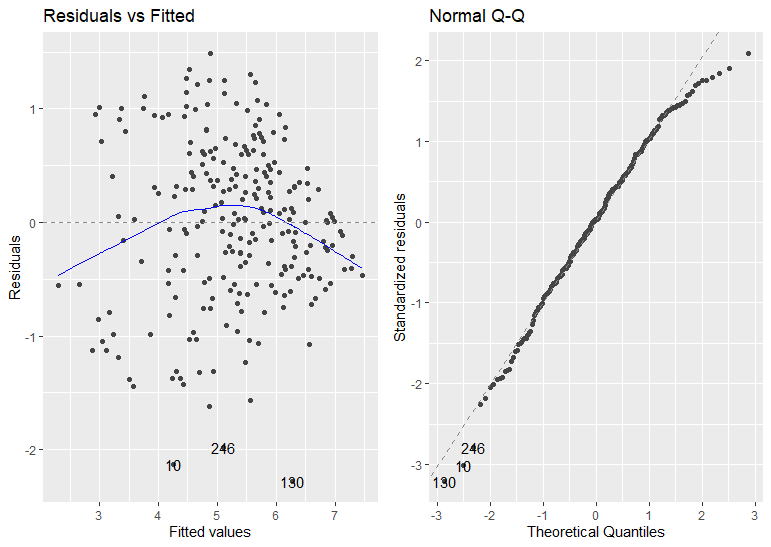
*

*Positivity*

*
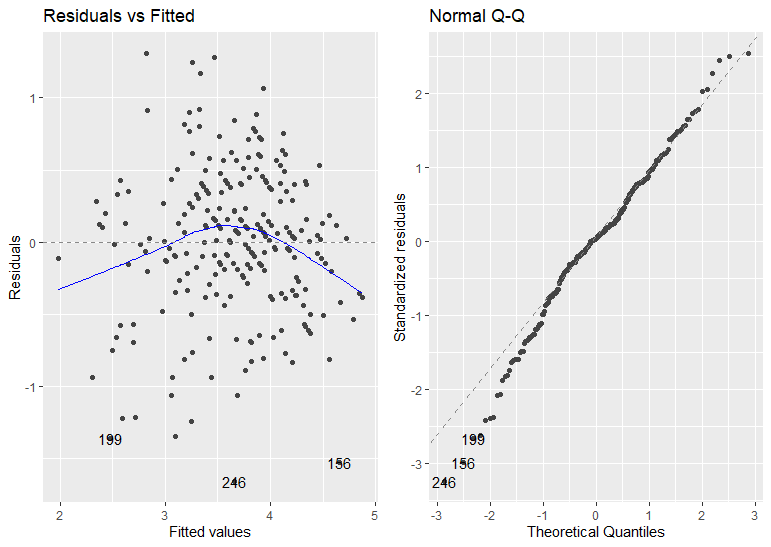
*

*Positivity with interaction*

*
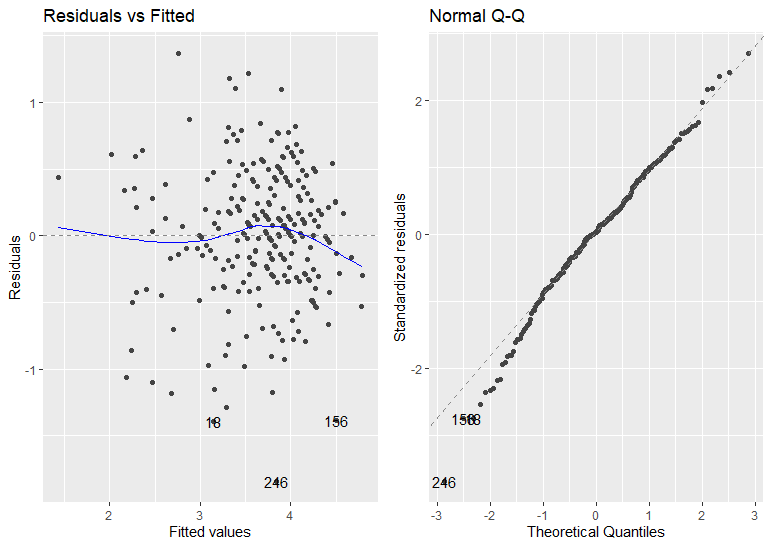
*
